# Mycelia as a focal point for horizontal gene transfer among soil bacteria

**DOI:** 10.1038/srep36390

**Published:** 2016-11-04

**Authors:** Tom Berthold, Florian Centler, Thomas Hübschmann, Rita Remer, Martin Thullner, Hauke Harms, Lukas Y. Wick

**Affiliations:** 1 Helmholtz Centre for Environmental Research - UFZ, Department of Environmental Microbiology, Permoserstraße 15, 04318, Leipzig, Germany; 2 German Centre for Integrative Biodiversity Research (iDiv) Halle-Jena-Leipzig, Deutscher Platz 5E, 04103 Leipzig, Germany

## Abstract

Horizontal gene transfer (HGT) is a main mechanism of bacterial evolution endowing bacteria with new genetic traits. The transfer of mobile genetic elements such as plasmids (conjugation) requires the close proximity of cells. HGT between genetically distinct bacteria largely depends on cell movement in water films, which are typically discontinuous in natural systems like soil. Using laboratory microcosms, a bacterial reporter system and flow cytometry, we here investigated if and to which degree mycelial networks facilitate contact of and HGT between spatially separated bacteria. Our study shows that the network structures of mycelia promote bacterial HGT by providing continuous liquid films in which bacterial migration and contacts are favoured. This finding was confirmed by individual-based simulations, revealing that the tendency of migrating bacteria to concentrate in the liquid film around hyphae is a key factor for improved HGT along mycelial networks. Given their ubiquity, we propose that hyphae can act as focal point for HGT and genetic adaptation in soil.

Natural soils are buzzing with microscopically small life forms, since the complex structure of soil provides numerous microhabitats creating a plethora of ecological niches. Bacteria play a dominant role, with typical densities of 10^8^ cells per gram of soil and estimates reaching 10^5^ bacterial species in a single gram of soil^1^,^2^. They constantly have to adapt to changing environmental conditions, such as fluctuating availabilities of substrates and nutrients or exposure to toxic compounds. Besides using their phenotypic plasticity, bacteria are capable of transferring single functional genes or whole gene clusters within and between species. Such horizontal gene transfer (HGT) is a main driver of the evolution of bacteria^3^,^4^. Conjugation, i.e. the direct transfer of mobile genetic elements such as plasmids from a donor to a recipient cell, is considered to be the most important HGT mechanism^5^,^6^. Conjugation in soil is influenced by various factors such as donor-recipient compatibility, nutrient availability, temperature and pH; spatial isolation of cell aggregates and biofilm patches, and the restricted mobility of bacteria in the porous soil environment impede bacterial conjugation^7^,^8^,^9^,^10^,^11^,^12^. Active movement of bacteria is restricted by the limited connectivity of surfaces and discontinuity of water phases. Bacterial displacement over longer distances thus requires episodes of water flow or bioturbation^13^. Mycelial organisms like fungi establish a dense network of thread-like hyphae, which provides ample amounts of nutrients and colonisable surfaces that both are prerequisites for bacterial HGT^14^. Plasmids of ecological relevance have been discovered in the vicinity of hyphae, such as plasmid-encoded genes for monoaromatics degradation in *Pseudomonas fluorescens*^15^ or elevated levels of IncP-1β plasmids, known to encode a variety of ecological traits^16^,^17^. Starting out from these observations, we hypothesized that mycelia promote bacterial conjugation by acting as transport networks for bacteria, a concept described as the ‘*fungal highway*’^18^,^19^,^20^. Specifically, we tested if mycelial networks help conjugation partners to overcome the barrier represented by air-filled pores and if such ‘*fungal highways*’ constitute contact zones for bacteria with the overall effect of enhanced HGT.

We used a reporter system for bacterial Horizontal Gene Transfer, based on motile *Pseudomonas putida* strains, revealing HGT events by an emerging green fluorescence signal. To test a possible bridging effect of mycelial networks, plasmid donor and recipient were inoculated on individual agar pieces separated by an air-filled gap. Environments allowing for low, medium and high mobility of bacteria were simulated using (semi-)solid media prepared with varying agar concentrations^20^ and HGT events evaluated by flow cytometry and microscopy. The effect of transport networks was studied by adding either mycelia or glass fibres as abiotic surrogates to exclude other than physical effects.

## Results

### Quantification in laboratory microcosms

**Figure 1:**
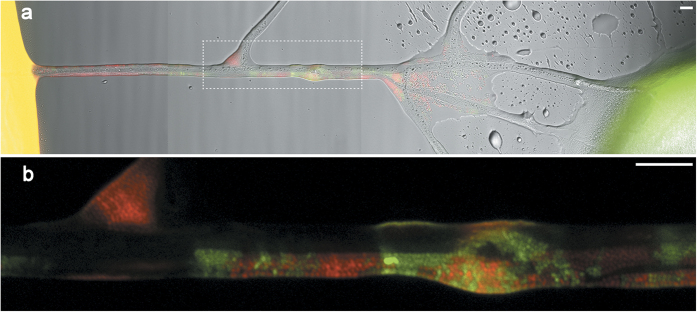
*Pseudomonas putida* donor and wildtype cells traversing an air gap along a mycelium of *Pythium ultimum*. (**a**) Combined epifluorescence and transmission light image showing mycelium of *P. ultimum* grown between separate agar pieces inoculated with donor (red) or wildtype (colourless) cells, respectively. Emerging transconjugants (green) are visible along the mycelium. Outlined area is shown in detail in (**b)**. **(b**) Combined image of red and green fluorescence channels showing the arrangement of donor and transconjugant cells along the mycelial segment. All scale bars represent 10 μm.

The role of hyphal structures of mycelia on bacterial HGT was studied by applying plasmid donor strain *Pseudomonas putida* KT2442::*dsRed-lacI*^*q*^(pWW0::*P*_*lac-*_*gfp*), which expresses the red fluorescent protein DsRed and the repressor LacI^q^, inhibiting the expression of a plasmid-encoded GFP. *Pseudomonas putida* KT2440 acted as potential plasmid recipient; the resulting transconjugants *Pseudomonas putida* KT2440(pWW0::*P*_*lac*_*-gfp*) express GFP indicating successful plasmid transfer^21^. To simulate an air-filled soil pore, donor and wildtype were inoculated on agar pieces separated by an air gap of about 400 μm. The oomycete *Pythium ultimum* was inoculated on the donor side. Within 24 hours it formed a mycelium bridging the gap thereby establishing a viable transport path between both bacterial populations (Fig. 1). The presence of all three cell types – donor, wildtype and resulting transconjugant – in the vicinity of the hyphae was visualized by epifluorescence microscopy. In control setups consisting of separated systems without mycelial structures, no transconjugants were detected.

**Figure 2:**
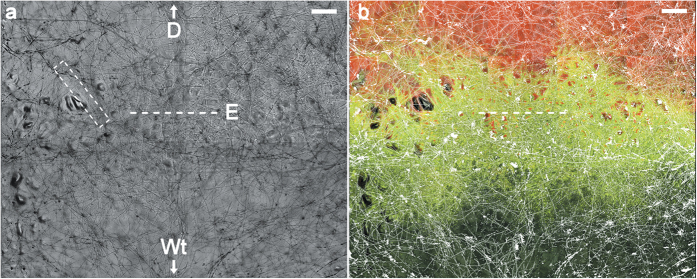
Localisation of *Pseudomonas putida* transconjugants in systems with mycelial networks. (**a**) Transmission light image of agar surface with mycelial network of *P. ultimum*. White arrows indicate inoculation points of *P. putida* KT2440 wildtype-strain (Wt) and KT2442 donor-strain (D) in a distance of 7 mm on the established mycelial network. Zone in the dashed white box was used as an input for the simulation model in Fig. 4a. The dashed white line represents the equal distance between both inoculation points (E). (**b**) Combined image of red and green fluorescence channels with an overlay of the mycelial structure shown in white. Transconjugants can be seen emerging along the network structures. Scale bars represent 500 μm. Images were taken 3 days after incubation.

HGT frequencies in systems with or without transport networks were quantified on (semi-) solid ABC-media^21^ containing varying amounts of agar to influence bacterial mobility. High mobility agar containing 0.3% w/v agar and medium mobility agar (0.5% w/v) allowed for almost unhindered or hampered swimming motility^19^,^20^,^22^, respectively, whereas low mobility agar (1.8% w/v) strongly restricted bacterial movement. The media were either overgrown with *P. ultimum*, overlaid with glass fibres or left uncovered. The aforementioned plasmid donor strain and *Pseudomonas putida* KT2440::*yfp* as potential recipient strain were inoculated at a separation distance of 7 mm on a continuous agar surface and the systems incubated for three days. The resulting transconjugants in this case constitutively express YFP and additionally started expressing GFP, allowing them to be differentiated via microscopy and quantified by flow cytometry^21^. After the incubation, the mycelia-covered area between the inoculation spots of donor and recipient colonies displayed numerous transconjugants (Fig. 2). The HGT promoting effect of the mycelium was especially notable at the equidistant contact zone between donor and recipient colonies (Fig. 2). Here, the transconjugants appeared along and in between the hyphal network structures of *P. ultimum* thereby acting as ‘hot spots’ for plasmid transfer.

**Figure 3:**
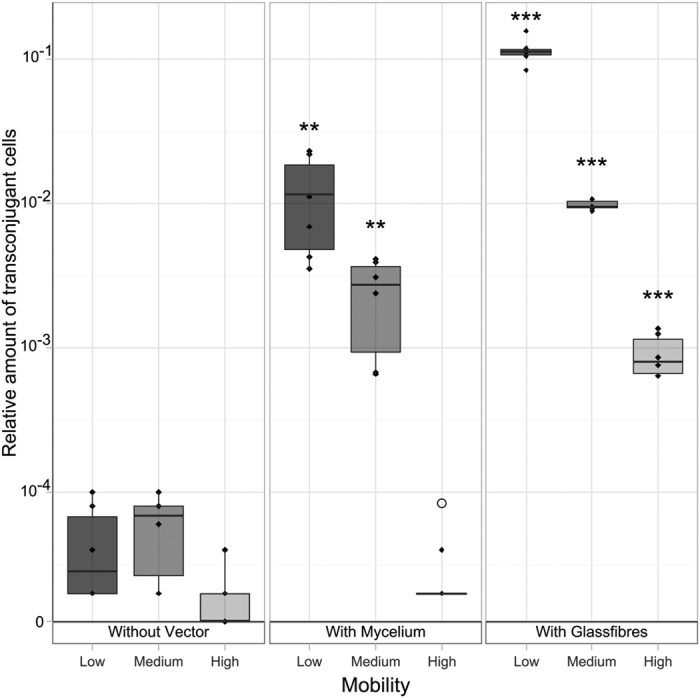
Relative amount of transconjugants in the microcosm setups after 3 days of incubation (n = 6). Diamonds represent single data points; black lines show arithmetic mean, grey boxes show upper and lower quartile. Removal of outliers detected with Grubbs’ test (α = 0.05) shown as circle. Absolute zero values shown as “0” in the otherwise logarithmic scale. Significant differences to respective systems without vectors were determined by Students’ t-test. (**p < 0.01; ***p < 0.001).

In plain agar systems without transport vectors, only very few transconjugants (relative amount: 1–6 × 10^−5^ transconjugant cells) were observed, even in high mobility systems that permitted bacterial swimming (Fig 3). While comparably low transconjugant numbers were likewise found in high mobility systems with mycelia (2.4 × 10^−5^ cells), a significantly higher amount of transconjugants was detected in mycelial systems permitting medium or low mobility (2.49 and 11.87 × 10^−3^ cells, respectively, two-sided Student’s t-test with p values of 0.0078 and 0.0032). Thus, the beneficial effect of mycelia was especially pronounced in low and medium mobility systems, i.e. in environments where bacteria had to rely on the mycelial structures for migration and habitat colonization. The same trend was even more pronounced in systems supplemented with glass fibres as abiotic, i.e. metabolically inactive transport vectors. In presence of glass fibres, systems showed significantly higher amounts of transconjugants than without transport vectors in all three cases (relative transconjugant fractions of 1.15 × 10^−1^, 9.79 × 10^−3^ and 9.2 × 10^−4^ cells for low, medium and high mobility systems, respectively, two-sided Student’s t-test with p values of 4.12 × 10^−7^, 5.03 × 10^−11^ and 3.09 × 10^−5^). Even in high mobility systems with glass fibres there was a notable increase in transconjugants, compared to systems with mycelia or without transport vectors. This increase is attributed to the fact that the glass fibres represent ‘ideal’ transport vectors: straight, unbranched transport pathways with a hydrophilic surface, creating wide continuous water films for bacterial movement, while at the same time providing a rigid surface for bacteria to settle and conjugate.

### Individual-based model simulation

**Figure 4:**
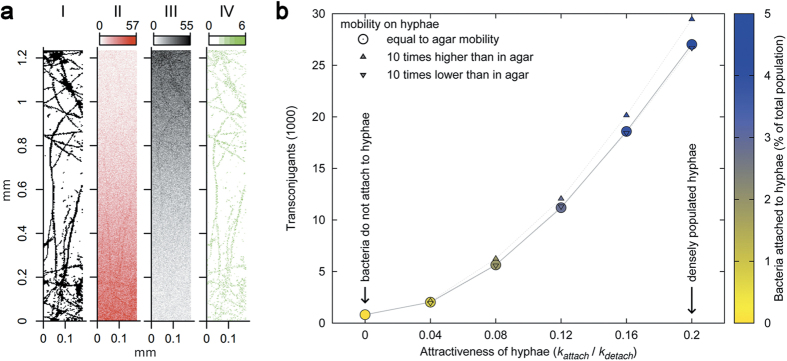
Simulation results of (**a**) the spatial distribution of transconjugants and (**b**) the role of hyphal attractiveness on conjugation rate in low mobility systems with mycelia. (**a**) hyphal network used in the simulation model (derived from a microscopic image, see Fig. 2a) (I), simulated distribution of donor (II) and recipient (III) cell numbers, and cumulative spatial distribution of conjugation events (IV) after 2.5 minutes of simulated time (halfway through total simulation time). Parameter values were *D*_*agar*_ = *D*_*hyphae*_ = 1.5 × 10^−5^ cm^2^/s, *k*_*attach*_ = 0.1 s^−1^, *k*_*detach*_ = 0.5 s^−1^. (**b)** simulated number of transconjugants after 5 minutes of simulation time for different attractiveness levels of hyphae for bacteria. Attractiveness levels of hyphae (defined by changing *k*_*attach*_ while keeping *k*_*detach*_ = 0.5 s^−1^) ranged from low attractiveness, as in high mobility agar systems where bacterial mobility was unhindered, to high attractiveness systems, such as low mobility agar, in which the bacteria had to rely on mycelial networks to explore the system. As attractiveness increases more bacteria populated the hyphal network (color) and more conjugation events occured. Cases in which hyphal networks hindered (▼) or promoted bacterial mobility (▲) had only a minor impact. *D*_*agar*_ was set to 1.5 × 10^−5^ cm^2^/s.

To further investigate the underlying effects, an individual-based model was employed (cf. Supplementary Information for details). Model parameters were chosen according to the laboratory microcosm or literature values except for the characterization of the exchange flux of bacterial cells between the agar phase and the liquid film on the hyphal surface. This flux, defined by the attachment and detachment parameters *k*_*attach*_ and *k*_*detach*_, was systematically varied, in effect allowing bacterial cells to utilize the hyphal network to different extents. Additionally, single cell mobility along the hyphae was varied relative to the cell’s mobility in agar. As bacterial migration in our planar experimental setup is mainly following along a horizontal plane, either within the agar layer or along the flat hyphal network on top the agar, we discretized the agar layer as a two-dimensional grid on which cells as part of opposing fronts of donor- and recipient cells randomly explore the system. The structure of a mycelium of *P. ultimum* overgrowing an agar surface (marked in Fig. 2b to 2a), was used as the hyphal network and placed as a second layer on the conceptual agar plane in our model, making our simulation quasi three-dimensional. During the simulated time of 5 minutes which was sufficient for both bacterial fronts (initially 1.2 mm apart) to mix well, conjugation events mainly occurred along the hyphal networks (Fig. 4a) and, hence, confirm experimental observations (Fig. 2b). In order to assess the drivers for the HGT promoting effect of hyphae, a detailed analysis of the proposed driving parameters of HGT along hyphae was performed. It revealed that the extent of transconjugants depended on the attractiveness of the hyphae for bacteria (k_attach_/k_detach_), which determined both the bacterial fraction colonizing the hyphal surface and the number of transconjugants (Fig. 4b). Interestingly, conjugation rates depended only to a minor extent on the relative bacterial mobility on hyphal surfaces relative to the agar (Fig. 4b).

## Discussion

In this study we give experimental and theoretical evidence that mycelia-based dispersal promotes HGT due to an elevated contact probability between bacteria. Conjugation requires close physical contact between bacterial donor and recipient cells with reported effective distances of <2 μm for *Pseudomonas putida*^21^,^23^ and typical periods between 3.5–4.0 min^24^ in which plasmid donor and recipient remain idle next to each other^25^. Here, we show for the first time that the narrow liquid films along continuous surfaces of hyphae and glass fibres promote close cell-to-cell contact by increased settling of initially spatially separate conjugation partners along the network structures^10^ (Fig. 3). While previous microbial conjugation models assumed well-mixed populations^26^,^27^ or considered space explicitly in up to three dimensions^28^, we used a quasi three-dimensional model to corroborate our findings.

We propose two driving mechanisms for the increased contact probabilities in the hyphosphere: Reduction of the space available for bacterial dispersal and, secondly, reduction of the degree of freedom of bacterial mobility. Such reasoning becomes clear by comparing a scenario in which bacterial cells populate a cube of water with a scenario in which cells only populate a water film surrounding hyphae forming a network within a cube of the same volume. Even in case of dense networks, the latter scenario results in a clearly reduced volume available for bacterial dispersal. In contrast to the water cube scenario, hyphae additionally represent defined corridors for dispersal, and cells migrating along a hypha can overcome longer linear distances than in the water cube in a given time span. Presuming bacterial mobility within the liquid film around hyphae resembles a two- rather than a three-dimensional movement, the calculated increase of contact probability of two cells amounts to a factor of approximately 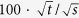 (see Supplement for derivation); i.e. that the contact probability in the lower-dimensional domain increases over time (*t*) and surpasses the probability in the higher-dimensional domain by two orders of magnitude already after a very short time (~1 s). This effect may become even larger if thin water films reduce the randomness of bacterial movement^29^ or even prevent a reversal of the direction of movement, i.e. lead to virtually one-dimensional movement of cells^30^. Indeed, fewer transconjugants were detected when transport networks were added to high mobility systems; here hyphae are losing their exclusivity as transport paths and their ability to confine the movement of the cells with the effect of reduced frequencies of bacterial encounter and HGT. Simulation experiments corroborated the effect. They indicated that the volume reduction effect leading to higher bacterial densities in the vicinity of mycelial networks is the dominant cause for increased HGT activity in the experimental system.

### Relevance of the hyphosphere for bacterial HGT

Filamentous fungi represent up to 75% of the subsurface microbial biomass (0.2–0.4 mg g_dry soil_^−1^) with networks extending to 10^2^ to 10^4^ m length per g of topsoil^31^ and some networks reported to spread over hundreds of hectares^32^. Mycelial organisms have been shown to internally translocate compounds from nutrient-rich to nutrient-poor regions; thereby not only facilitating their own growth^33^,^34^, but also allowing surrounding microorganisms to thrive on exuded compounds^35^. By connecting air-filled space between water-filled pores^36^, fungi also provide efficient dispersal networks (‘fungal highways’^19^) for random or directed extra-hyphal movement of otherwise immobilised bacteria^37^. Mycelia thereby facilitate the access of bacteria to suitable microhabitats for growth^20^, enable efficient contaminant biodegradation^38^ or increase the functional stability in systems exposed to osmotic stress^39^.

Fungi, in particular symbiotic mycorrhizal fungi, are prevalent dwellers of the root zone of plants and may be a key driver for the ‘rhizosphere effect’ in HGT^40^, attributed to high cell densities in the mycosphere^14^ in response to fungal exudates or the chemotaxis-driven colonization^41^. Our data emphasize the physical role of mycelia in enabling bacterial transport and concentration. While three-dimensional water-filled space also permits high bacterial mobility, it does not offer the spatial confinement of mycelial networks penetrating low mobility environments, which act as ‘scaffold’ for bacterial movement and HGT^10^. The concentrating effect of mycelia will be particularly pronounced when the liquid film associated with the mycelium represents a substantial part of the habitable space; for instance in unsaturated soil or when mycelium-associated liquid films are likely to gather bacteria in situations of receding soil water, thus concentrating bacteria in the hyphosphere.

Xerophilic fungi are furthermore able to grow at low water activities of only 0.65^42^ and, more importantly, unlike bacteria, which are immobilized at matric potentials of <−3.6 kPa^43^, fungi do not require continuous water phases for active dispersal. Earlier studies have shown that bacterial dispersal along mycelia depends both on the physico-chemical surface properties of the hyphae and the motility of the bacteria: Although most effective dispersal was found by flagella-driven swimming along hydrophilic mycelia^19^, transport of non-flagellated bacteria along hydrophilic hyphae^18^,^44^,^45^ or of flagellated bacteria along more hydrophobic surfaces has been described^19^. It is reasonable to assume that the hyphal benefit for bacterial HGT is not restricted to the ideal situation chosen in this experiment, yet rather may vary depending on the bacteria and fungi and their possible antagonistic effects^46^.

The role of mycelia as preferential dispersal pathways and promotors of HGT between bacteria hence also may be a driving factor of the evolution of bacterial diversity and may have led to significant increase of prokaryotic diversity after the appearance of the mycelial fungi in microbial evolution.

## Materials and Methods

### Microbial strains

A bacterial reporter system consisting of two *Pseudomonas* strains was used to visualize HGT events as described by Seoane^21^. *Pseudomonas putida* KT2442::*dsRed-lacI*^*q*^(pWW0::*P*_*lac*_*-gfp*) was used as plasmid donor, expressing DsRed while at the same time repressing plasmid-encoded GFP by the simultaneous expression of LacI^q^. *Pseudomonas putida* KT2440::*yfp* acted as potential plasmid recipient, constitutively expressing YFP^21^. The resulting transconjugant *Pseudomonas putida* KT2440::*yfp*(pWW0:: *P*_*lac*_*-gfp*) simultaneously expresses YFP and GFP, detectable with appropriate filter sets for yellow and green fluorescence, indicating successful plasmid transfer. Donor cells were grown on Brunner-Medium^47^ supplemented with 10 mM *m*-toluic acid (mTol) to select for the pWW0 plasmid. Recipient and wildtype cells were grown on R2A medium^48^.

Transconjugant cells were created by cross-streaking donor and recipient cells on R2A media supplemented with 20 mM mTol and 18 g/L agar and cultivation for 24 hrs at 30 °C. After selection for plasmid harbouring cells on Minimal-Medium-Agar with 20 mM mTol, colonies were microscopically checked for their fluorescence signals. Transconjugant cells were selected based on the presence of yellow and green fluorescence and the absence of red fluorescence. Presence of the plasmid in the transconjugant cells was confirmed by plasmid isolation and subsequent gel electrophoresis, as described in the Supplementary Information.

The oomycete *Pythium ultimum* was grown at room temperature on plates of AB-medium^21^ supplemented with 10 mM Na-citrate (ABC) and 18 g/L agar.

### Epifluorescence microscopy

For epifluorescence microscopy, the *P. putida* KT2442 donor strain was used in combination with the *P. putida* KT2440 wildtype-strain as potential plasmid recipient. To observe the influence of mycelial networks on spatially isolated donor and wildtype colonies, individual ABC agar pieces (18 g/L agar) were inoculated with 1 μL overnight cultures adjusted to an optical density of 0.05. As mycelial organism, *P. ultimum* was additionally inoculated on the donor side. The agar pieces were inverted and placed side by side in a 50-mm glass bottom dish (Ibidi, Munich, Germany) with an air gap of about 400 μm between them. Setups were incubated at room temperature for 3 days and examined with an AZ100M fluorescence microscope (Nikon, Düsseldorf, Germany), using distinct filtersets for DsRed (F46-005) and GFP (F46-002, both AHF, Tübingen, Germany). Images were captured with the Nikon NIS-Elements software and annotated with Adobe Photoshop (Adobe Systems, San Jose, CA, USA).

### Microcosm Setups

Depending on the agar concentration, the used media generally permitted three dimensional movements of motile bacteria. Liquid (3 g agar/L) and semi-solid agar (5 g agar/L) were assumed to allow nearly unhindered and hampered movement, respectively, whereas movement in solid agar (18 g agar/L) was regarded negligible^13^,^38^.

To investigate the effect of alternative transport routes provided by mycelial networks, microcosms were set up in triplicates in 12-well plates (Greiner Bio-One, Frickenhausen, Germany) by filling each well with 2 mL AB-medium supplemented with 10 mM Na-citrate and the agar concentrations of choice. Solid and semi-solid agar were left to dry for 24 h, liquid agar for 1 h. *P. ultimum* was inoculated on the side of each well and incubated at room temperature for 3 days until a complete, planar mycelial network had established on the agar in each well. Setups with abiotic transport networks were prepared in parallel by using glass fibres with diameter of ≈12 μm, cut to a length of 2 cm (P-D Glasseiden, Oschatz, Germany). Before covering the agar with approximately 300 fibres for each well, the glass fibres were cleaned by heating to 600 °C for 5 h^49^. All wells were subsequently inoculated with 1 μL each of overnight cultures of donor and recipient, adjusted to an optical density of 3.75 at a separation distance of 7 mm. After incubation for 3 days at room temperature, each microcosm was resuspended in 2 mL of phosphate buffered saline (PBS) to harvest the cells by vortexing and ultrasonication as described by Kohlmeier *et al*.^19^. Flow cytometry was used to quantify the cells according to their fluorescence signal.

To microscopically observe the influence of the mycelial network on transconjugant formation in the microcosms, parallel setups with a combination of *P. putida* donor and wildtype strain were examined after 3 days of incubation. Images were taken with an AZ100M fluorescence microscope (Nikon, Düsseldorf, Germany), using the NIS-Elements software and distinct filtersets for DsRed (F46-005) and GFP (F46-002, both AHF, Tübingen, Germany). Adobe Photoshop (Adobe Systems, San Jose, CA, USA) was used to annotate images and reduce noise. To reveal localization of transconjugants along mycelial network structures, a white overlay of the mycelial network was created by applying a signal intensity threshold to a transmission light image and modest manual corrections.

### Flow Cytometric Analysis

Flow cytometric measurements were carried out using a MoFlo cell sorter (DakoCytomation, Fort Collins, CO, USA) equipped with a water-cooled argon-ion laser (Innova 70C, Coherent, Santa Clara, CA, USA). 488 nm light of 400 mW was used to analyze the forward light scatter (FSC) and the side light scatter (SSC), and to excite GFP, YFP, and dsRed. Fluorescences were detected after passage of the optical filters 510/10 (GFP), 542/27 (YFP), and 600/37 (dsRed), respectively (Semrock Inc., Rochester, NY, USA). Data recording and subsequent data analyses were made applying Summit software v4.3 (DakoCytomation, Fort Collins, CO, USA).

For absolute quantification, cell numbers per mL were determined using reference microspheres (FluoSpheres polystyrene microspheres, 1.0 μm, yellow-green fluorescent, 505/515, Molecular Probes, Eugene, OR, USA) of known concentration as described previously^50^. Prior to measurement, the cell suspensions were filtered through a 50-μm CellTrics filter (Sysmex Partec, Görlitz, Germany) to exclude debris.

The identification and quantification of GFP/YFP fluorescent transconjugants within a cell suspension containing a multitude of YFP fluorescent recipient and dsRed fluorescent donor cells was done as follows: Pure cultures of recipient, donor, and transconjugant cells were analyzed by flow cytometry. On an FL1 (GFP fluorescence) vs. FL2 (YFP fluorescence) dot plot, three areas (gates) were defined with the premises that gate 1 exclusively includes transconjugant cells, gate 2 exclusively those of the recipient strain, and gate 3 those of the FL1/FL2 non-fluorescent donor strain. Based on this definition, all conjugational samples were analyzed. If a cell had displayed a gate 1 specific FL1/FL2 fluorescence it was classified as a transconjugant. The total number of transconjugants per sample was calculated by means of the above mentioned yellow-green fluorescent, 1 μm microspheres.

The transconjugant cell numbers within each replicate series (n = 6) were tested for skewness^51^ and outliers were detected using Grubbs’ test (α = 0.05). The results of the replicate series for low, medium, and high mobility systems without transport networks were compared to the respective systems with transport networks, using a two-sided Student’s t-test to test for significant differences (significance levels: *p = 0.05; **p = 0.005; ***p = 0.001).

### Individual-based Model

Microbial cells populated a two-dimensional grid of two layers in the model. The bottom layer was continuous and represented the agar, while the top layer was obtained by digitizing a microscopic image of actual fungal hyphae to represent the hyphal network. Microbial cells performed random walks (as implemented earlier^52^) on both layers with prescribed diffusion constants *D*_*agar*_ and *D*_*hyphae*_, and could switch layers at locations where a fungal grid cell was present on top the agar grid cell according to


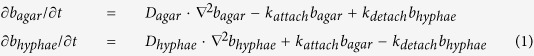


with *b*_*agar*_ and *b*_*hyphae*_ denoting microbial cells on the respective layer and rate parameters *k*_*attach*_ and *k*_*detach*_ describing bacterial attachment and detachment from fungal hyphae. Following Levin^26^, plasmid transfer between the plasmid bearing donor population *X*_*D*_ and plasmid-free recipient population *X*_*R*_ giving rise to the transconjugant population *X*_*T*_ was modeled within individual grid cells according to


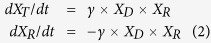


with rate parameter γ^53^. The model was implemented as a stochastic, individual-based model, initialized with a population of donor cells on one domain boundary and a population of recipient cells on the opposing boundary, with keeping cell numbers constant along both boundaries during the simulation. A total simulation time of 5 minutes was enough for population fronts to mix well, but short compared to the typical lag-phase between transconjugant events. Hence, all cells were allowed to only take part in one HGT event, requiring to consider only three cell types in the model: donor cells, recipient cells, and HGT-wise inactive cells. Further modeling details are provided in the Supplementary Information. The simulation source code (in Standard C) is available from the authors on request.

## Additional Information

**How to cite this article**: Berthold, T. *et al*. Mycelia as a focal point for horizontal gene transfer among soil bacteria. *Sci. Rep.* **6**, 36390; doi: 10.1038/srep36390 (2016).

**Publisher’s note:** Springer Nature remains neutral with regard to jurisdictional claims in published maps and institutional affiliations.

## Supplementary Material

Supplementary Information
