# Supplementary material for: Mycelia as a focal point for horizontal gene transfer among soil bacteria
Source: Sci Rep. 2016 Nov 4;6:36390. doi: 10.1038/srep36390 (PMC5095653; doi:10.1038/srep36390)
Supplement: Supplementary Information [file srep36390-s1.pdf]

Mycelia as a focal point for horizontal gene transfer among soil bacteria

Berthold, Tom[a]; Centler, Florian[a]; Hübschmann, Thomas[a]; Remer, Rita[a]; Thullner, Martin[a]; Harms, Hauke[a,b]; Wick, Lukas Yvo[a*]

[a] *Helmholtz Centre for Environmental Research - UFZ, Department Environmental Microbiology, Permoserstraße 15, 04318 Leipzig, Germany*

[b] *German Centre for Integrative Biodiversity Research (iDiv) Halle-Jena-Leipzig, Deutscher Platz 5E, 04103 Leipzig, Germany*

*Corresponding author: Lukas Y. Wick

E-mail: lukas.wick@ufz.de

Supplementary Information

Plasmid isolation

The presence of the plasmid was confirmed by using a modified alkaline lysis protocol for the isolation of large plasmids (A. M. Boronin, personal communication):

An overnight culture of the *Pseudomonas* strains was harvested by centrifugation and resuspended in 75µl 25mM Tris-HCl(pH 7.0). After addition of 75µ STET solution (16% (w/v) Sucrose, 10% (v/v) Triton X-100, 0,1M EDTA (pH8.0), 0,1M Tris-HCL (pH7.0), alkaline lysis was initiated by adding 300µl 0.2N NaOH and storing the suspension on ice for 15 minutes. After addition of 300µl 2M Tris-HCl and 300µl 10M LiCl, the suspension was kept on ice for 60 minutes before centrifugation and a subsequent washing step of the DNA pellet with 70% (v/v) Ethanol. The DNA was dried, resuspended in 30µl TE Buffer (10mM Tris-HCl (pH 7.5), 1mM EDTA (pH 8.0)) and visualized on a 0.8% agarose gel (Supplementary fig. 1).

Theoretical derivation of contact probabilities

Direct cell contact is a prerequisite for microbial horizontal gene transfer (HGT). Probabilities for such contacts depend on the degrees of freedom microbial cells experience with respect to motility. Cells populating a water cube, for example, effectively explore a three-dimensional space while cells populating a thin film of water, for example surrounding mycelial hyphae, effectively only explore a two-dimensional area. To evaluate the impact on contact probabilities, we consider a continuous water medium allowing for free random bacterial movement, which is thought to be three-, two-, or one-dimensional. Considering a particle, or bacterial cell, performing a random walk and starting at the point of origin, the probability of this particle to reside at time t within a specific domain Ω_x is given by the

integral of the diffusion equation with diffusion constant D and written for d dimensions over this domain:

$$\overline{P_d}(\Omega_x, t) = \int_{\Omega_x} \frac{1}{(4\pi D t)^{\frac{d}{2}}} e^{-\frac{|x|^2}{4Dt}}. \qquad \text{(Eq. 3)}$$

Depending on the considered dimensionality d, the domain Ω_x refers to a volume, an area, or an interval with its centre located at x. The size δ of this domain is chosen so that if two particles populate the same domain they can be expected to have physical contact, for example by choosing the typical diameter of the bacterium as δ. If a second particle starts its random walk in an initial distance of l from the first particle, the probability $\overline{P_d^C}$ for a contact of both particles in a given domain at time t is then given by the product of the probabilities for both particles to reside in that same domain Ω_x:

$$\overline{P_d^C}(\Omega_x, \mathrm{t}) = \overline{P_d}(\Omega_x, \mathrm{t}) \times \overline{P_d}(\Omega_{x-l}, \mathrm{t}). \qquad \text{(Eq. 4)}$$

To compute the probability $P_d(t)$ that a contact happens anywhere at time t, space needs to be decomposed into a sequence of non-overlapping domains Ω_i of short lateral length δ. Taking the sum over contact probabilities for all these domains yields

$$P_d(t) = \left[\frac{\delta}{2\sqrt{2\pi D t}}\right]^d \cdot e^{-\frac{l^2}{8Dt}}. \qquad \text{(Eq. 5)}$$

The effect of decreasing the dimensionality, for example from three to two dimensions is then given by $P_{d-1}(t) = P_d(t) \cdot \lambda$ with $\lambda = \left[\frac{2\sqrt{2\pi D t}}{\delta}\right]$. For typical values of δ and D (δ = 2 µm, $D = 1.5 \times 10^{-5}$ cm^2/s), λ amounts to to $97.1 s^{-0.5} \cdot \sqrt{t}$, indicating that the contact probability in

the lower-dimensional domain increases over time and surpasses the probability in the higher-dimensional domain by two orders of magnitude after short time (1 s).

Symbolic calculations were performed using Mathematica 9 (Wolfram Research Inc., IL, USA).

Individual-based model

An agar section of 4.2 mm height, comparable to the experimental setup, covering a surface of approximately 0.17 mm × 1.2 mm was horizontally discretized into square microhabitats of 3.523 µm lateral length, resulting in a two-dimensional grid of 50 × 350 microhabitats representing the agar domain in the model. A representative section of a microscopic image of actual fungal hyphae was discretized at the same resolution and added as a second layer in the model as the hyphal domain. Hyphal microhabitats where assumed to have a depth of 1µm, only supporting a single layer of bacterial cells.

Random bacterial movement in both the agar and the hyphal domain was simulated as Fickian diffusion with a fixed diffusion constants D_{agar} and D_{hyphae} [cm^2/s], allowing cells to explore the continuous agar space and the transport network provided by the fungal hyphae. Microbial cells were allowed to migrate between the agar and hyphal domain with prescribed rate parameters k_{attach} [s^{-1}] for hyphal attachment and k_{detach} [s^{-1}] for hyphal detachment. Two bacterial fronts, one consisting of donor cells and the other consisting of recipient cells, were migrating towards each other in the model. Plasmid transfer was assumed to take place within both agar and hyphal microhabitats and to depend on both donor concentration X_D [cells / ml] and recipient concentration X_R [cells / ml] within the respective microhabitats. The transfer rate was given by $\gamma \times X_D \times X_R$ with rate parameter γ [ml/cell/min][41]. The total simulation time of 5 minutes allowed for both bacterial fronts to

mix well, was longer than the presumed duration of plasmid transfer between bacteria[52], but was shorter than the reported average lag phase between two transfer events from the same donor cell of 10-20 min[21]. Microbial cells were therefor allowed to only take part in one HGT event during the simulation.

All processes including HGT, transfer between domains, and random cell motility within domains were simulated stochastically, requiring rates to be expressed as probabilities. For example, the probability for a donor cell to transfer a plasmid to a recipient cell within a simulation time step of length Δt is given by $\gamma \times X_R \times \Delta t$. Similarly, the probability for a hyphae-attached cell to detach is given by $k_{detach} \times \Delta t$. Cell motility was implemented as described previously[52]. A time step size Δt of 2 ms was used.

Initially, 25 recipient cells were placed in each agar habitat along the top of the simulation domain boundary and 25 donor cells in each agar habitat along the bottom domain boundary, resulting in cell concentrations of 0.48×10^6 cells/µl which was comparable to the inoculated cell concentration used in the experiment (ca. $0.23 - 1.0 \times 10^6$ cells/µl). During the simulation, cells were added along these boundaries as necessary to maintain this concentration. According to the scanned image of the actual hyphal network, the top and bottom boundaries featured a different number of hyphal microhabitats. This leads to a difference in accessibility of the network for bacteria entering the simulation domain from both sides. To minimize these effects, we added hyphal microhabitats along the full extent of both boundaries in the model.

For plasmid transfer, a rate constant of $\gamma = 3.3 \times 10^{-12}$ ml/cell/min was selected as has been reported for plasmid transfer in *Pseudomonas aeruginosa*[53]. For bacterial cell motility in agar, a diffusion constant of $D_{agar} = 1.5 \times 10^{-5}$ cm^2/s was selected as a typical value for swimming agar. For motility along hyphae, the diffusion constant D_{hyphae} was set to the same

value, or to values one order of magnitude above or below to evaluate the impact of inhibited or enhanced motility along hyphae. The ratio of the attachment and detachment rate parameters k_{attach}/k_{detach} determines the equilibrium distribution of cells between the agar and the hyphal domain. To avoid overpopulated hyphal habitats, a maximum ratio of 0.2 was selected which corresponds to 25 cells residing in the agar and 5 in the hyphal habitat in equilibrium, selecting $k_{attach} = 0.1s^{-1}$ and $k_{detach} = 0.5s^{-1}$. To assess the importance of the hyphal network for HGT, this ratio was also lowered by choosing smaller values for k_{attach}, leading to fewer bacterial cells populating the hyphal network. Model results showed little stochastic variability when repeating simulations, caused by the large number of bacterial cells considered. While the ratio k_{attach}/k_{detach} determines the attractiveness of the hyphal network for bacteria, leading to lightly or densely populated networks, the magnitude of the individual parameters indicate the transfer activity of bacterial cells between the agar and the hyphal network. We varied this intensity by multiplying both parameters with the same factor *f*, thus keeping the ratio unchanged, covering three orders of magnitude above and below the reference values (Supplementary fig. 2). The magnitude of the exchange activity of bacteria switching between agar and the hyphal network had no effect for $k_{attach} \geq 0.1\ s^{-1}$. Only for $k_{attach} < 0.1\ s^{-1}$, for which the simulation time of 5 minutes was not enough to equilibrate the agar and hyphal populations, an increase in transconjugants occurred (Supplementary fig. 2).

We note that while the agar domain is defined to be of 4.2 mm height, it is not guaranteed that cells take advantage of the entire available volume, as is assumed in the model. Rerunning simulations with smaller effective agar volumes revealed however, that even for an effective height of 1mm, hyphal conjugation events were still dominating in all tested scenarios except for small hyphal attractiveness levels with $k_{attach}/k_{detach} = 0.04$.

Supplementary Figures

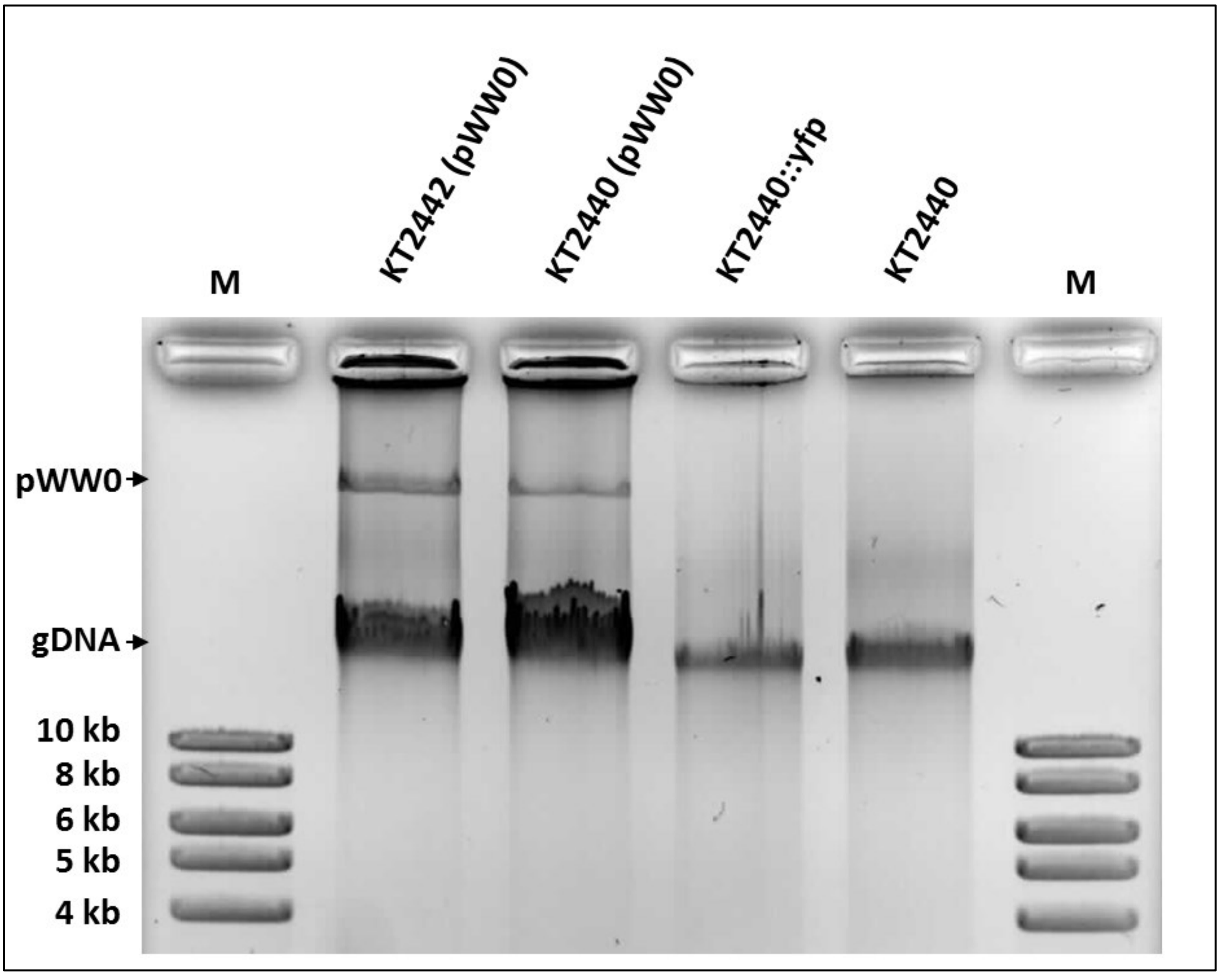

Supplementary Figure 1: Comparison of plasmid extractions of the *Pseudomonas putida* strains used in this study. Sample order from left to right: Donor strain *Pseudomonas putida* KT2442::*dsRed-lacI*^{q}(pWW0::P_{lac}-*gfp*), transconjugant strain *Pseudomonas putida* KT2440::*yfp*(pWW0::P_{lac}-*gfp*), yfp-labeled recipient strain *Pseudomonas putida* KT2440::*yfp*, unlabeled recipient strain *Pseudomonas putida* KT2440. 1 kb DNA Ladder (NEB, Ipswich, MA, USA) was used as DNA marker. In the donor and transconjugant lanes, the pWW0 plasmid band is visible above the genomic DNA.

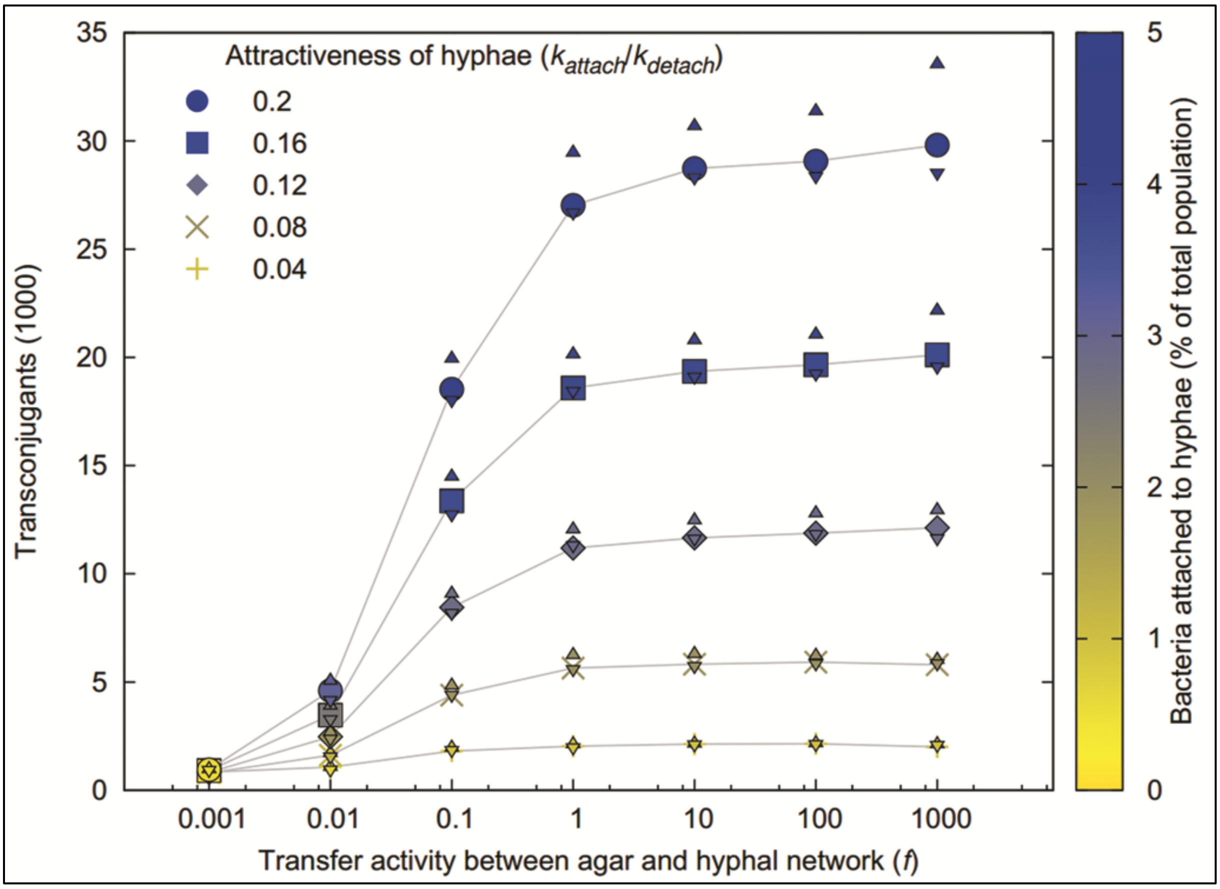

Supplementary Figure 2: Simulated number of transconjugants after 5 minutes of simulation time for different attractiveness levels of hyphae for bacteria (k_{attach} /k_{detach}) and different transfer activities (setting k_{attach} = $f \times k_{attach,ref}$ and k_{detach} = $f \times k_{detach,ref}$; reference values for k_{attach} /k_{detach} = 0.2 were $k_{attach,ref}$ = 0.1s^{-1} and $k_{detach,ref}$ = 0.5s^{-1}, reference values for other ratios were obtained by changing k_{attach}). D_{agar} and D_{hyphae} were set to 1.5 × 10^{-5} cm^2/s. Small triangles show results for setting D_{hyphae} = D_{agar} × 10 (▲) and D_{hyphae} = D_{agar} / 10 (▼).
